# Hereditary breast and ovarian cancer triggered by occult fallopian tube cancer: a case report

**DOI:** 10.1186/s13256-023-04095-6

**Published:** 2023-08-18

**Authors:** Hikaru Murakami, Satoe Fujiwara, Ruri Nishie, Shoko Ueda, Shinichi Terada, Takashi Yamada, Masahide Ohmichi

**Affiliations:** 1 Department of Obstetrics and Gynecology, Osaka Medical and Pharmaceutical University, 2-7 Daigakumachi, Takatsuki, Osaka 569-8686 Japan; 2 Department of Pathology, Osaka Medical and Pharmaceutical University, 2-7 Daigakumachi, Takatsuki, Osaka 569-8686 Japan

**Keywords:** Occult cancer, HBOC, Occult metastasis to para-aortic lymph node

## Abstract

**Background:**

At the time of benign gynecological surgery, a prophylactic salpingo-oophorectomy or salpingectomy is increasingly being performed concurrently to reduce the risk of future ovarian and fallopian tube cancer. We herein describe a case of hereditary breast and ovarian cancer syndrome in which a hysterectomy and bilateral adnexectomy were performed with a preoperative diagnosis of benign tumor. A detailed pathological examination revealed occult fallopian tube cancer, and additional staging surgery provided an accurate pathology diagnosis.

**Case presentation:**

A 72-year-old Japanese woman with a past history of breast cancer underwent a hysterectomy and bilateral oophoro-salpingectomy for the preoperative diagnosis of uterine myoma and a right para-ovarian cyst. In the detailed pathological examination, high-grade serous carcinoma of the right fallopian tube was detected incidentally, and a subsequent staging laparotomy confirmed single para-aortic lymph node metastasis. Furthermore, a mutation in germline *BRCA*2 was detected postoperatively, and the patient was finally diagnosed with hereditary breast and ovarian cancer syndrome. She was diagnosed with fallopian tube cancer International Federation of Gynecology and Obstetrics Stage IIIA1(i) and started on adjuvant therapy (six courses of paclitaxel and carboplatin followed by maintenance therapy with olaparib), and 18 months after surgery, she was free of disease.

**Conclusion:**

This is a case of fallopian tube cancer that was diagnosed incidentally and then accurately staged with additional advanced staging surgery. Even in the absence of grossly malignant findings, a detailed pathological search of the fallopian tubes and accurate staging surgery are important to make the necessary treatment decisions for the patient.

## Background

When the resection of ovaries or fallopian tubes is performed for patients with a preoperative diagnosis of benign gynecological tumor, occult cancer may be revealed incidentally. When ovarian or fallopian tube cancer is diagnosed unexpectedly, additional staging surgery and *BRCA* testing are important in determining the patient’s course of postoperative treatment or surveillance. In previous reports, accurate pathology diagnosis has been lacking in a number of cases due to the lack of additional staging surgeries. We herein describe a case of hereditary breast and ovarian cancer syndrome (HBOC) in which a hysterectomy and bilateral adnexectomy were performed with a preoperative diagnosis of benign tumor; a detailed pathological examination revealed occult fallopian tube cancer, and additional staging surgery provided an accurate pathology diagnosis.

## Case presentation

**Fig. 1:**
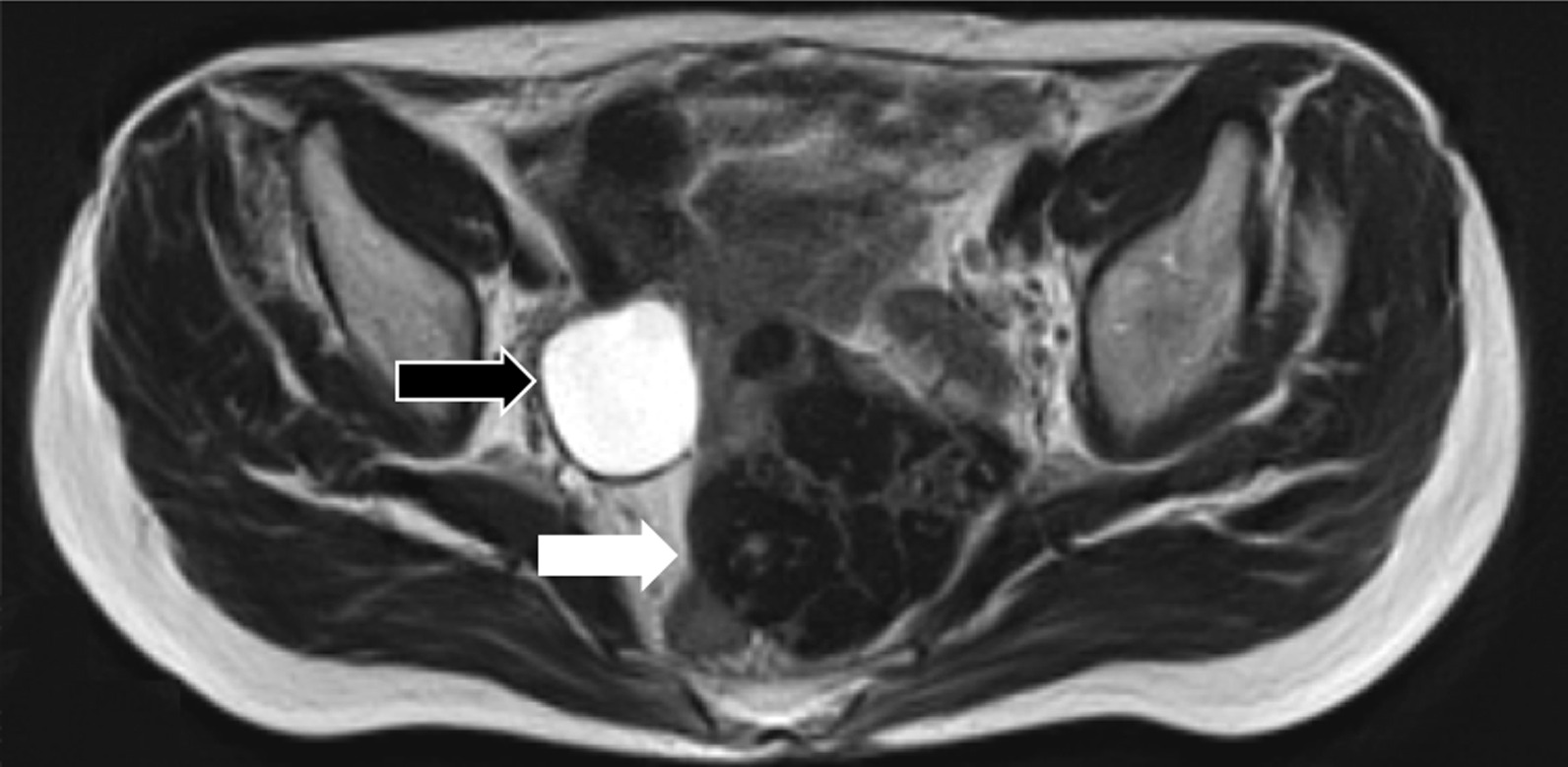
Magnetic resonance imaging of the pelvis. T2-weighted image reveals an 8 cm low-density tumor (white arrow) located at the posterior surface of the uterus and a 5 cm high-density simple cyst (black arrow) located at the right para-ovary and without solid compartment

**Fig. 2:**
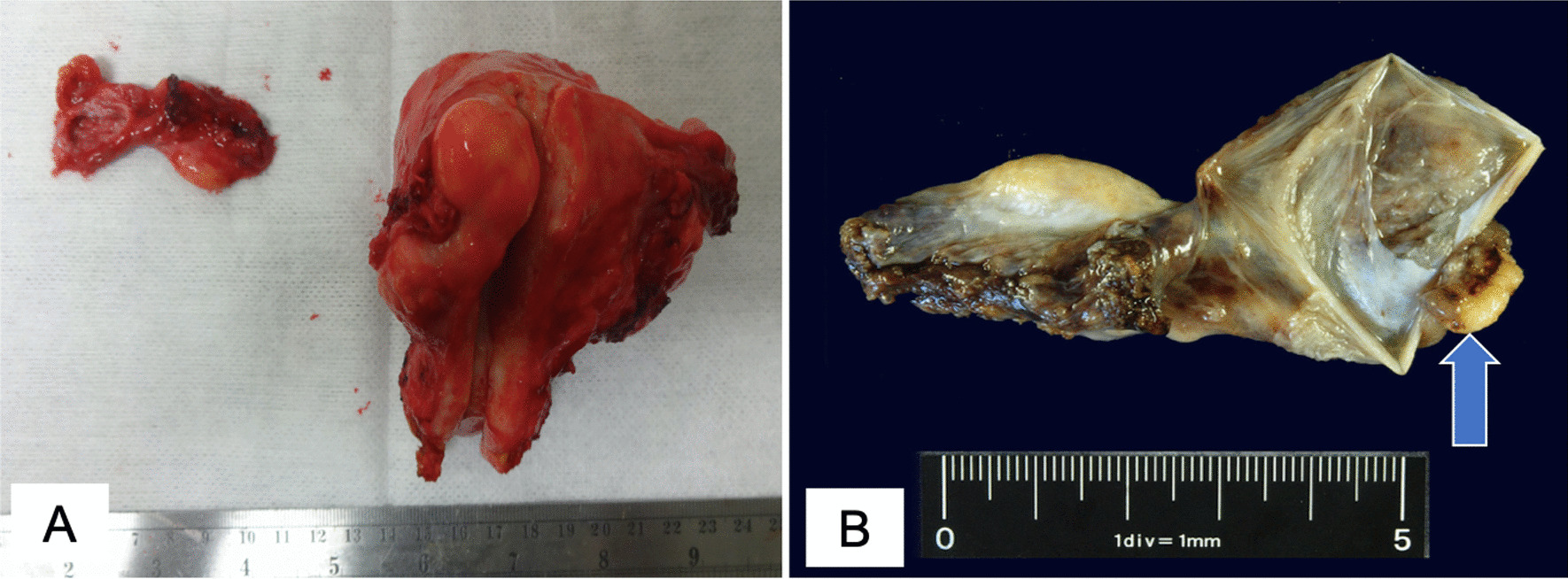
**A** Gross appearance of the resected specimens. Uterine myoma and right paraovarian cyst, but no gross abnormalities in the right fallopian tube. **B** Gross appearance of the right oophoro-salpinx after formalin fixation. A 5 mm-sized tumor is observed (arrow)

**Fig. 3:**
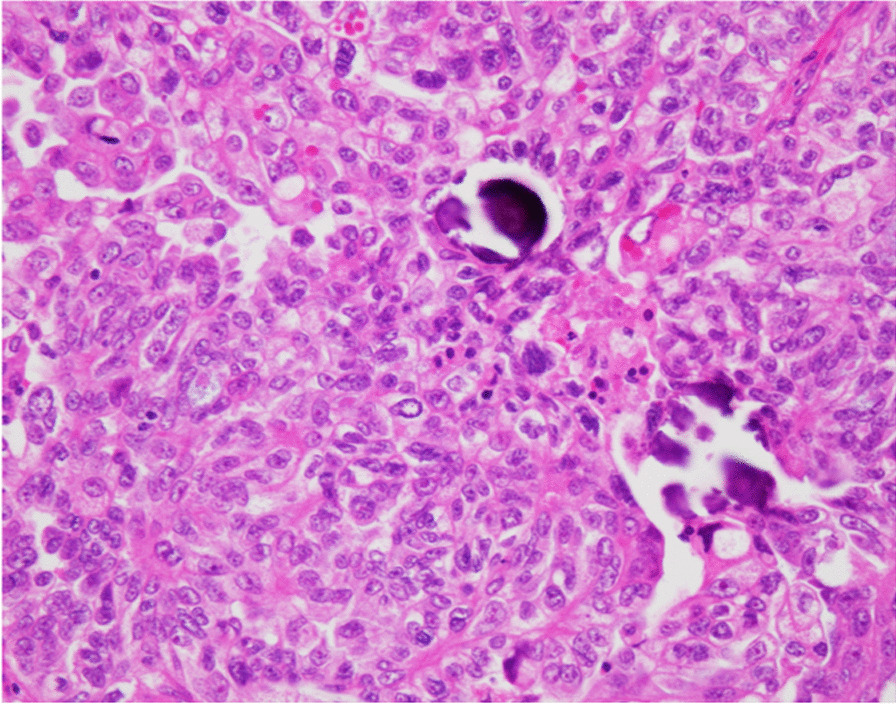
Microscopic appearance of the resected right fallopian tube. Hematoxylin and eosin staining. A 5 mm-sized epithelial tumor is observed in the right fallopian tube, showing papillary and alveolar forms, and solid areas and psammoma bodies are found. Nuclear atypia is strong, and many fission figures are recognized, which is consistent with high-grade serous carcinoma (× 400)

The patient was a 72-year-old Japanese woman who underwent breast-conserving surgery for left breast cancer when she was 47-years-old, followed by radiation therapy and tamoxifen treatment. There were no family histories suggesting HBOC. She presented to her previous physician with symptoms of frequent urination. An magnetic resonance imaging (MRI) scan revealed a uterine myoma located in the posterior wall of the uterus and a right para-ovarian cyst without any malignant findings (Fig. 1). Her preoperative serum level for cancer antigen 125(CA125), cancer antigen 19-9 (CA19-9), and carcinoembryonic antigen (CEA) were within their respective normal ranges. An abdominal simple total hysterectomy and bilateral oophoro-salpingectomy were performed to correct her frequent urination symptoms, which were pressure symptoms caused by the uterine myoma. There were no abnormalities in her right fallopian tube on preoperative imaging and gross examination (Fig. 2A, B). The pathological examination revealed a uterine myoma and a right para-ovarian cyst with no evidence of malignancy. However, a 5 mm-sized tumor showing papillary and alveolar forms, along with solid areas and psammoma bodies, was observed in the right fallopian tube. Nuclear atypia was strong, and many mitoses were recognized. Immunostainings were positive for PAX-8, p16, WT-1, and p53, and negative for Napsin A and CEA, thus consistent with high-grade serous carcinoma (Fig. 3). With the diagnosis of primary fallopian tube cancer, she was referred to our hospital for additional treatment of the disease.

The patient’s serum level for CA125, CA19-9, and CEA were within their respective normal ranges at our hospital, as well, and an additional computed tomography (CT) scan showed no enlarged lymph nodes nor distant metastases. Our preoperative diagnosis was primary right fallopian tube cancer, which was suspected at International Federation of Gynecology and Obstetrics (FIGO) stage IA, and a staging laparotomy, including a pelvic and para-aortic lymphadenectomy, partial omentectomy, peritoneal biopsy, and ascites cytology, was performed. There were no macroscopical lesions in the abdominal cavity and no enlarged lymph nodes in both the pelvic and para-aortic areas. The postoperative course was uneventful.

**Fig. 4:**
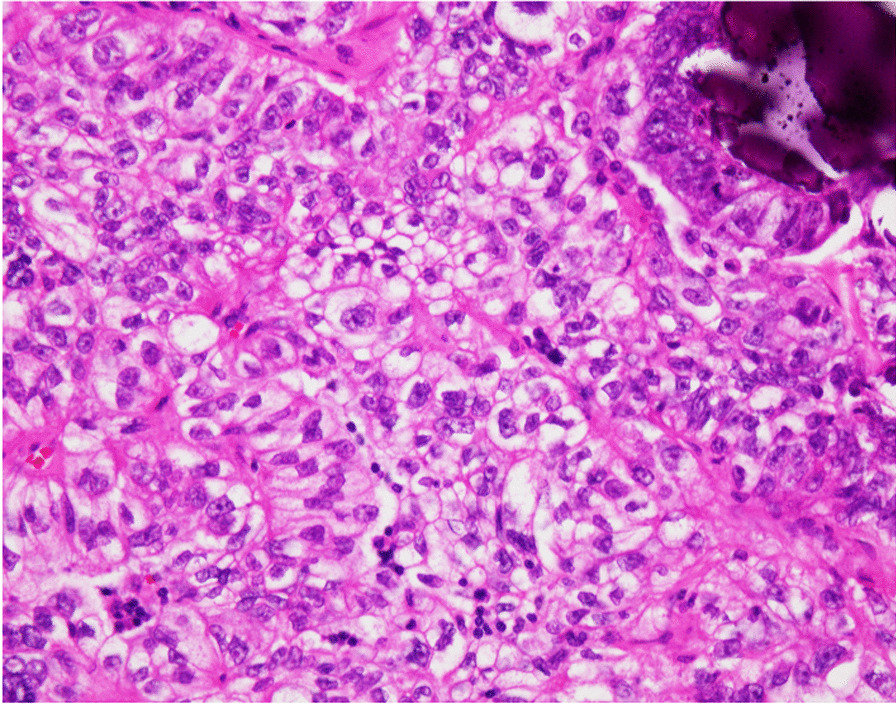
Microscopic appearance of the resected para-aortic lymph node. Hematoxylin and eosin staining. Similar to the image seen in the right fallopian tube, solid areas and psammoma bodies are found in the tumor with strong nuclear atypia, which is consistent with metastasis of right fallopian tubal high-grade serous carcinoma (× 400)

A postoperative pathological examination revealed a sporadic metastasis of less than 10 mm in the para-aortic lymph node. Histology of the metastatic tumor was similar to the tumor in the right fallopian tube, and solid areas and psammoma bodies were found with strong nuclear atypia. Immunostainings were positive for AE1/AE3, CK7, PAX-8, p16, WT-1, and p53 and negative for CK20, thus being consistent with the metastasis of right fallopian tubal high-grade serous carcinoma (Fig. 4) (pelvic lymph node 0/20, para-aortic lymph node 1/17). On the other hand, the ascites cytology was negative, and no metastasis was found in the omentum, peritoneum, and the pelvic lymph nodes. With the final diagnosis of right fallopian tube cancer stage IIIA1(i) (FIGO 2018), high-grade serous carcinoma (HGSC), pT1aN1M0, six courses of dose-dense TC (paclitaxel 80 mg/m^3^/week, carboplatin AUC6/3 weeks) were administered intravenously as adjuvant chemotherapy. Owing to the patient’s diagnosis of fallopian tube cancer, as well as her previous history of breast cancer, it was strongly suspected that she had HBOC. Although she initially refused genetic testing, a *BRCA* test was performed on her after we explained that it was an important test for therapeutic drug selection and surveillance. The *BRCA* testing revealed that she has a germline mutation in *BRCA2*.

Based on the results of her genetic testing, the patient started six courses of paclitaxel and carboplatin, followed by maintenance therapy with olaparib for 2 years. She was free of disease 18 months after surgery.

## Discussion

A risk-reducing salpingo-oophorectomy (RRSO) for patients with hereditary breast and ovarian cancer syndrome has been shown to be effective in preventing the development of ovarian cancer [1]. An RRSO analysis has concluded that high-grade serous carcinoma (HGSC) originate from the epithelium of the fallopian tube rather than from the ovary [2–4]. Genetic abnormalities in the fallopian tube epithelium are also reported to lead to the development of the fallopian tube epithelium from serous tubal intraepithelial carcinoma (STIC), a precursor lesion, to HGSC [2–4]. Therefore, a salpingectomy is considered to be useful for the prevention of ovarian cancer [5]. The concept of STIC recommends an opportunistic salpingectomy as a strategy for epithelial ovarian cancer prevention, and a bilateral salpingectomy is increasingly being performed in surgery for gynecological disease, even when there are no gross lesions.

Several previous reports have shown that occult cancers were found in the fallopian tubes with a certain degree of frequency when a pathological examination was performed postoperatively. When gynecological surgery is performed for benign tumors, it is reported that the frequency of finding occult cancer is 1.44% for endometrial cancer, 0.60% for cervical cancer, and 0.19% for ovarian cancer. The frequency of ovarian cancer found as an occult cancer was reported to be 0.04% in those under 40 years old, 0.15% in those aged 40–54 years, and 0.47% in those aged 55 years and over [6]. Thus, the incidence of occult ovarian cancer increases with advancing age, but most cases are detected at an early stage [7]. Fallopian tube cancer, which has a lower incidence than ovarian cancer, is even less likely to be found as an occult cancer. Some have suggested that a pathological examination may not be accurate and stress the need for a precise examination. In our case, no macroscopic abnormalities could be pointed out from the pre- and intraoperative examination; however, the pathological examination revealed a region of high-grade serous carcinoma in the right fallopian tube fimbriae. In this case, the lesion in the fallopian tube sheath was very small, measuring only 5 mm. It is assumed that the diagnosis of fallopian tube cancer may be missed in some cases due to similarly minute lesions. The examination for fallopian tubes that appear to be grossly normal also requires a detailed exploration, according to the sectioning and extensively examining the fimbriated end (SEE-FIM) protocol, as does the evaluation of specimens at RRSO for HBOC [8].

According to the National Comprehensive Cancer Network’s (NCCN) Guidelines for ovarian cancer (2023), it is recommended to consider surgical staging if not previously done for staging laparotomy for patients with newly diagnosed ovarian cancer and to determine the treatment after surgery, observation, or systematic chemotherapy [9]. On the other hand, if a staging laparotomy is not chosen, adjuvant chemotherapy is recommended, assuming that there is potential residual disease [9–11]. It has been suggested that adjuvant chemotherapy, in the absence of an adequate staging laparotomy, may improve the patient’s prognosis. However, these reports were followed by the efficacy of maintenance therapy including poly ADP-ribose polymerase (PARP) inhibitors based on genetic testing. Since 2018, several phase 3 studies, such as the SOLO-1, PRIMA, and PAOLA-1, have demonstrated the efficacy of maintenance therapy with PARP inhibitors after adjuvant chemotherapy for patients with advanced ovarian cancer, especially in those with a *BRCA* mutation [12–14]. If an adequate staging laparotomy is not performed, the necessary maintenance therapy may not be provided, and the patient may not achieve the expected prognostic effect. Young *et al*. reported that, in ovarian cancer, occult stage III is present in 23% of cases that are presumed to be stage I grossly, due to the presence of microscopic lymph node metastasis [15]. Baekelandt *et al.* also reported that the frequency of lymph node metastasis was higher in fallopian tube cancer than in ovarian cancer, with retroperitoneal lymph node metastasis in 40–60% of cases, and a similar frequency of metastasis to the pelvic and para-aortic lymph nodes [16].

In this case, we judged that reoperation was feasible based on the patient’s condition, so we reoperated and performed a staging laparotomy. There were no enlarged lymph nodes on preoperative and intraoperative examination, but the pathological examination revealed a solitary metastasis in the para-aortic lymph nodes. The final diagnosis of this patient was changed from FIGO stage IA (pT1aNxM0) to FIGO stage IIIA1(i) (pT1aN1M0) due to this staging laparotomy, thus indicating the need for maintenance therapy. Past reports have described cases in which staging surgery was omitted in patients in which ovarian cancer was found incidentally after surgery [17]. However, our case proved that a staging laparotomy in fallopian tube cancer is important because of the potential of metastasis to the peritoneal lymph nodes, even when the primary tumor is small and there are no enlarged lymph nodes. In addition, although this patient had no family history of breast or ovarian cancer, we strongly suspected undiagnosed HBOC due to her previous history of breast cancer after the diagnosis of fallopian tube cancer. The genetic examination revealed a g*BRCA2* variant. Therefore, olaparib maintenance therapy was started after six courses of adjuvant chemotherapy. Eighteen months after surgery, she was free of disease.

On the other hand, the patient initially refused to undergo a genetic examination. However, she eventually understood the importance of testing for treatment selection and risk management, for herself and for her family, and decided to undergo genetic testing. In Japan, the actual rate of testing when *BRCA* testing is offered is reported to be 6–51% [18], and in many cases, genetic testing is refused even in patients with a strong suspicion of HBOC, such as in our case. Risk management for ovarian and breast cancer in the individual and blood relatives for HBOC is important [19]. In particular, the effectiveness of RRSOs has been shown with regard to prevention of ovarian cancer development [1]. In addition, regarding the frequency of occult cancers found in the ovaries and fallopian tubes after RRSO performed in HBOC patients, including intraepithelial carcinomas, Yates *et al*. reported 7.9% [20], GOG0199 reported 2.6% [21], and Nomura *et al*. reported 2.6% in Japan [22]. The NCCN guidelines recommend that RRSO for HBOC should be performed by the age of 35–40 years for patients with the *BRCA1* mutation and by the age of 40–45 years for patients with the *BRCA2* mutation [9]. This patient was diagnosed and treated for breast cancer at the age of 47 years. If HBOC diagnosis had been made in the past and RRSO had been performed, the development of fallopian tubal cancer could have been prevented. The histological type of ovarian cancer in HBOC is often HGSC, as in this case, and is characterized by rapid metastasis [23]. Considering our HBOC case in which the fallopian tube cancer found in occult cancer had occult metastasis to the para-aortic lymph node, it is very important to perform a salpingo-oophorectomy before the onset of the disease, and the first step should be to perform RRSO at the recommended age. On the other hand, even if the patient is already beyond the recommended age for RRSO at the time of HBOC diagnosis, as in our case, there is a possibility of occult cancer with a risk of rapid progression to advanced cancer, thus indicating that prompt RRSO at the time of HBOC diagnosis may contribute to an improved prognosis. This requires appropriate genetic counseling.

## Conclusion

In conclusion, this is a case in which occult cancer was found in a fallopian tube that was not grossly abnormal, leading to the diagnosis of HBOC. Detailed evaluation of fallopian tube specimens removed during surgery for benign tumors is important to ensure that even microscopic lesions are not overlooked. We believe that accurate pathological diagnosis and adequate advanced-stage surgery are the most important factors to improve the prognosis of patients with fallopian tube cancer.

## Data Availability

The data used or analyzed are all included in this published article.

## Abbreviations

BRCA: Breast cancer susceptibility
HBOC: Hereditary breast and ovarian cancer
MRI: Magnetic resonance imaging
CA125: Carbohydrate antigen 125
CA19-9: Carbohydrate antigen 19-9
CEA: Carcinoembryonic antigen
CT: Computed tomography
FIGO: International Federation of Gynecology and Obstetrics
HGSC: High-grade serous carcinoma
TC: Paclitaxel carboplatin
AUC: Area under the concentration–time curve
RRSO: Risk-reducing salpingo-oophorectomy
STIC: Serous tubal intraepithelial carcinoma
SEE-FIM: Sectioning and extensively examining the fimbriated end
NCCN: National Comprehensive Cancer Network
PARP: Poly ADP-ribose polymerase
